# Multipolar mapping for catheter ablation of premature ventricular complexes originating from papillary muscles in the structurally normal heart: a case series

**DOI:** 10.1186/s12872-020-01747-z

**Published:** 2020-10-28

**Authors:** Chi Cai, Jing Wang, Hongxia Niu, Wei Hua, Jianmin Chu, Shu Zhang

**Affiliations:** The Cardiac Arrhythmia Center, State Key Laboratory of Cardiovascular Disease, Fuwai Hospital, National Center for Cardiovascular Diseases, Chinese Academy of Medical Sciences and Peking Union Medical College, Beilishi Road No. 167, Xicheng District, Beijing, 100037 China

**Keywords:** Papillary muscles, Premature ventricular complexes, Duodecapolar catheter, Catheter ablation

## Abstract

**Background:**

Previous studies on radiofrequency catheter ablation of premature ventricular complexes (PVCs) arising from the left ventricle (LV) papillary muscles (PM) show a modest procedural success rate with higher recurrence rate. Our study sought to explore the utility of using a multipolar mapping with a steerable linear duodecapolar catheter for ablating the PM PVCs.

**Methods:**

Detailed endocardial multipolar mapping was performed using a steerable linear duodecapolar catheter in 6 consecutive PM PVCs patients with structurally normal heart. The clinical features and procedural data as well as success rate were analysed.

**Results:**

LV endocardial electroanatomic mapping was performed in all patients via a retrograde aortic approach using a duodecapolar mapping catheter. All patients displayed a PVC burden with 16.2 ± 5.4%. Duodecapolar catheter mapping demonstrated highly efficiency with an average procedure time (95.8 ± 7.4 min) and fluoroscopy time (14.2 ± 1.5 min). The mean number of ablation applications points was 6.8 ± 1.9 with an average overall ablation duration of 6.1 ± 3.0 min. The values of earliest activation time during mapping using duodecapolar catheter were 37.8 ± 7.2 ms. All patients demonstrated acute successful ablation, and the PVC burden in all patients after an average follow-up of 8.5 ± 2.0 months was only 0.7%. There were no complications during the procedures and after follow-up.

**Conclusions:**

Mapping and ablation of PM PVCs using a duodecapolar catheter facilitated the identification of earliest activation potentials and pace mapping, and demonstrated a high success rate during follow-up.

## Background

Papillary muscles (PM) of the left ventricle (LV) are a source of premature ventricular complexes (PVCs) in patients with or without structural heart disease [1, 2]. Given their anatomic complexity and independent motion during the cardiac cycle, radiofrequency catheter ablation of PVCs arising from the PM has a fair procedural success rate and higher recurrence rate compared with other locations [3]. Because of the anatomical variability and the relative instability of the ablation catheter at these sites, successful point-by-point mapping to identify the ablation targets can be time-consuming and operator dependent. Recent studies have shown that high-density mapping with multipolar catheters has been shown to be accurate and expeditious in the mapping of complex reentrant atrial arrhythmias and scar-mediated ventricular tachycardias [4, 5]. Multipolar mapping may hold promise for facilitating an accurate and more efficient identification of ectopic foci in PM regions, thereby improving procedural success rate for PM PVCs ablation. Nevertheless, currently published studies regarding PM PVCs ablation using multipolar mapping approaches are still limited. The aim of this study is to present our experience with a multipolar mapping technique using a duodecapolar (20-electrodes) catheter (St. Jude Medical, St. Paul, MN, USA) for mapping and ablation of PM PVCs in a case series of patients with structurally normal hearts, and we sought to investigate the clinical features and procedural data as well as success rate after catheter ablation using the duodecapolar mapping technique.

## Methods

### Study population

This study investigated six consecutive patients with PVCs arising from the LV PM in whom ablation was performed using a retrograde aortic approach at Fuwai hospital between May and November 2019. All subjects met the following inclusion criteria: frequent symptomatic PVCs despite antiarrhythmic treatment and absence of underlying cardiomyopathy as well as mitral valve disease based on preprocedural echocardiography. Antiarrhythmic agents were discontinued for at least 5 half-lives before the procedure. All patients signed informed consent forms, and the study complied with the Declaration of Helsinki and was approved by the Institutional Review Board.

### Data collection

Data including demographics, echocardiographic parameters, 12-lead electrocardiograms (ECG), 24-h Holter monitoring and medications at the initial evaluation were retrospectively obtained from the electronic medical record. Echocardiography and 24-h Holter monitoring were prospectively performed before discharge as well as at 3, 6 and 12 months during follow-up in all patients through chart review. Arrhythmia recurrence was assessed by 12-lead ECG and 24-h Holter monitoring recordings.

### Echocardiographic evaluation

Transthoracic echocardiograms were obtained before and within 24 h following the ablation procedure as well as during final follow-up for all patients. Echocardiographic parameters including the left atrium diameter (LAD) and left ventricular end-diastolic diameter (LVEDD) were measured using a commercially available system (iE33; Philips Medical Systems) equipped with a 3.5-MHz transducer according with the recommendations of the American Society of Echocardiography protocols [6]. The left ventricular ejection fraction (LVEF) was calculated using the modified biplane Simpson’s rule from apical imaging planes. The severity of mitral regurgitation (MR) was assessed semi-quantitatively according to regurgitant area and orifice width using the color-flow Doppler images at the parasternal long-axis.

### Mapping and ablation procedure

The procedures were performed under local anesthesia without sedation. Local right groin anesthesia was performed with lidocaine 1%, 5–10 ml. A 3-dimensional electroanatomic mapping system (Ensite NavX velocity system, St Jude Medical) was used in all patients. Under fluoroscopy, the decapolar catheter with 5-mm electrodes and 2-mm interelectrode spacing was placed in the coronary sinus (CS) by the right internal jugular vein route, with the proximal poles located at the CS ostium. Right femoral artery access was subsequently established with an 8F sheath and anticoagulation with heparin was initiated to maintain a target activated clotting time of 250–350 s. Surface 12-lead ECG and intracardiac electrograms were recorded continuously with a speed of 100 mm/sec on LabSystem Pro (Bard Electrophysiology, Lowell, MA).

LV endocardial electroanatomic mapping was performed via the right femoral artery and longitudinally retrograde approach using a steerable linear duodecapolar catheter (Livewire, 2–2-2 mm spacing electrodes, St. Jude Medical, St. Paul, MN, USA) during sinus rhythm with spontaneous PVCs. It should be confirmed that the PVCs was not caused mechanically and that it coincided with the morphology of the spontaneous clinical PVCs during activation mapping. Following the completion of activation mapping, and once all earliest prepotential bipolar activity observed on the mapping catheter was tagged on the electroanatomic map, pace mapping was performed at these sites for comparison with the clinical PVCs 12-lead ECG template. Perfect pace map match was defined as a 12/12 lead match and good pace map match was defined as a 10/12 match.

Upon mapping completion, the duodecapolar catheter was replaced by an ablation catheter, through the retrograde approach with an open irrigated deflectable quadrupolar 3.5-mm-tip FlexAbility ablation catheter (St. Jude Medical, St. Paul, MN, USA), and ablation was performed at 30–40 W, temperature limit 45 °C @ 17 mL flow rate [7]. The targeted sites for ablation including sites exhibiting the earliest bipolar activity with a local unipolar QS pattern with a local activation preceding the QRS onset by ≥ 30 ms during PVCs combined with perfect/good pace maps, which were previously tagged on the electroanatomic map, should be reconfirmed by ablation catheter, and then the radiofrequency ablation application was delivered. The RF output was adjusted according to impedance drop and temperature at the operator’s discretion. The following parameters were recorded for each ablation application: average RF output, average impedance drop and duration of ablation. If the RF application elicited a suppression or elimination of the PVCs within the initial 30 s, the application was maintained for ≤ 120 s, targeting an impedance drop of 10–15 Ω or a diminution or abolishment of the local electrogram. Transthoracic echocardiography was instantly performed at the end of the ablation procedure to confirm the lesions formed on the PM. After the ablation procedure, intravenous isoproterenol (2–10 μg/min) and burst pacing from the RV was performed to assess arrhythmia inducibility. Acute procedural success was defined as abolition of either inducible or spontaneous clinical PVCs at the end of the ablation procedure. Clinical evaluation assessed by 24-h Holter monitoring recordings was performed at 3, 6 and 12 months follow-up.

### Statistics

Continuous data are presented as mean ± SD, and dichotomous data are expressed as numbers and percentages. Comparison of data between PVC burden pre and post ablation was performed using the paired samples *t* test. All analyses were performed with SPSS for Windows, version 19.0 (SPSS, Chicago, USA).

## Results

### Baseline characteristics

The demographics and clinical characteristics at baseline are summarized in Table 1. Of those, 2 (33.3%) were male. The average age was 31 ± 13 years and the average BMI was 22.1 ± 2.3 kg/m^2^. The whole study cohort had normal cardiac structure and function with a mean LVEDD of 45.2 ± 3.8 mm and LVEF of 63.5 ± 4.0%. All patients displayed a high PVC burden with 16.2 ± 5.4% and 5 (83.3%) presented with non-sustained ventricular tachycardia (NSVT). The QRS duration during PVCs was 145.2 ± 7.3 ms. All the patients received medical therapy before ablation, with β-blockers used in 83.3% of patients.

**Table 1:** Baseline demographic and clinical characteristics

Patient number	1	2	3	4	5	6
Demographics						
BMI, kg/m^2^	19.6	21.6	20.4	24.2	18.6	23.5
History of PVCs, months	60	9	36	6	84	6
Echocardiography						
LAD, mm	31	25	29	34	33	38
LVEDD, mm	45	43	44	46	41	52
LVEF, %	66	64	61	70	60	60
MR grade	Mild	None	None	None	Mild	Mild
ECG and 24 h-Holter ECG						
PVCs QRS duration, ms	152	144	134	140	148	153
PVC burden,%	24.1	13.3	17.1	20.8	11.5	10.7
NSVT	Yes	Yes	Yes	None	Yes	Yes
Medication treatments	Propafenone	β-Blocker; Mexiletine	β-Blocker	β-Blocker	β-Blocker	β-Blocker

The data are presented as the numbers (%) or the means. BMI, body mass index; PVCs, premature ventricular complexes; LAD, left atrial diameter; LVEDD, left ventricular end-diastolic diameter; LVEF, left ventricular ejection fraction; MR, mitral regurgitation; NSVT, nonsustained ventricular tachycardia

### Procedure and ablation characteristics

**Fig. 1:**
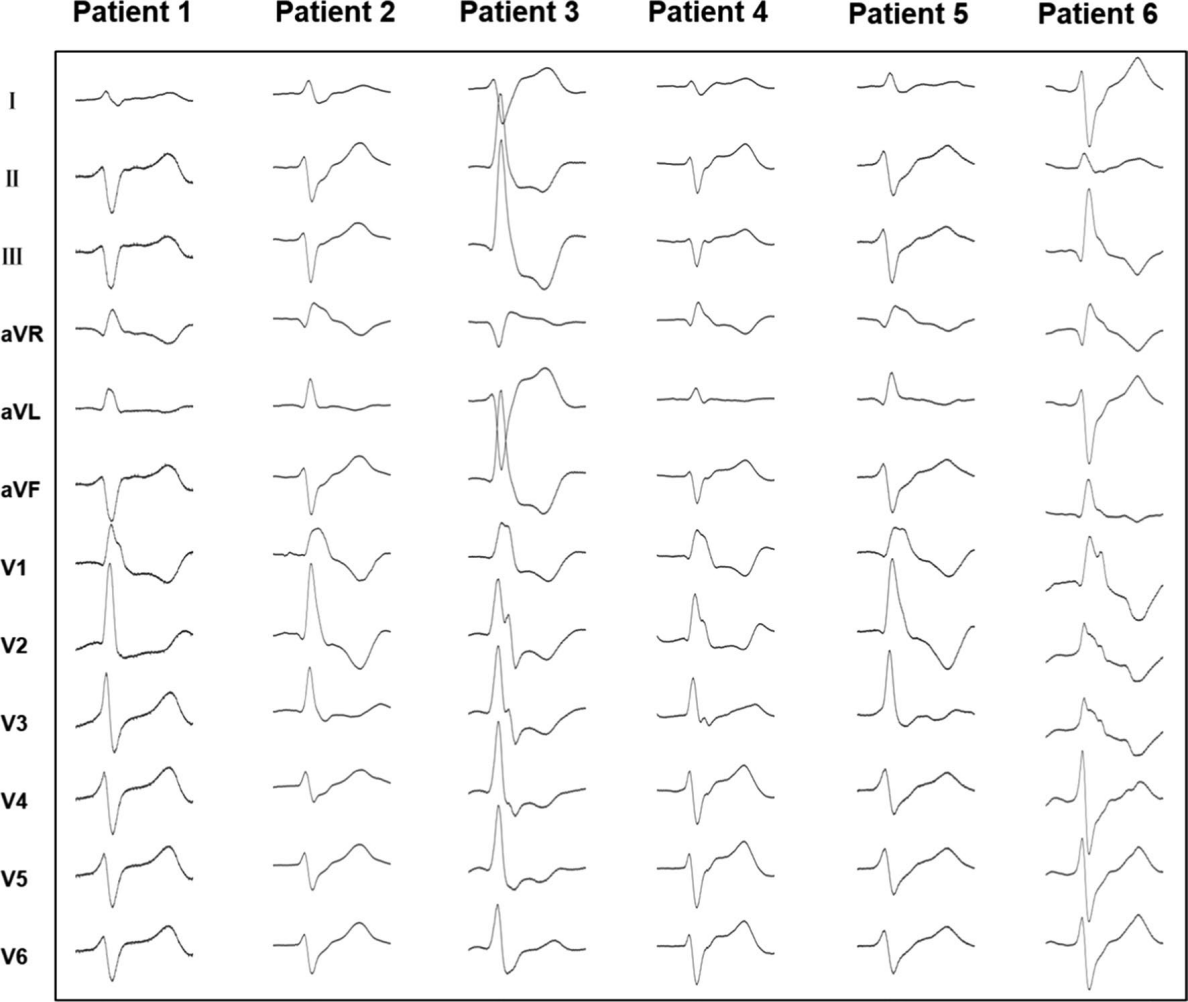
Twelve-lead electrocardiograms exhibiting PVCs arising from the APM and PPM. In patient 3 and 6, the PVCs have an RBBB pattern and right inferior axis, suggesting an origin at the APM. In patients 1, 2, 4 and 5, the PVCs morphology corresponds to an RBBB pattern and left superior axis, suggesting an origin at the PPM. PVCs, premature ventricular complexes; APM, anterolateral papillary muscle; PPM, posteromedial papillary muscle; RBBB, right bundle branch block

**Fig. 2:**
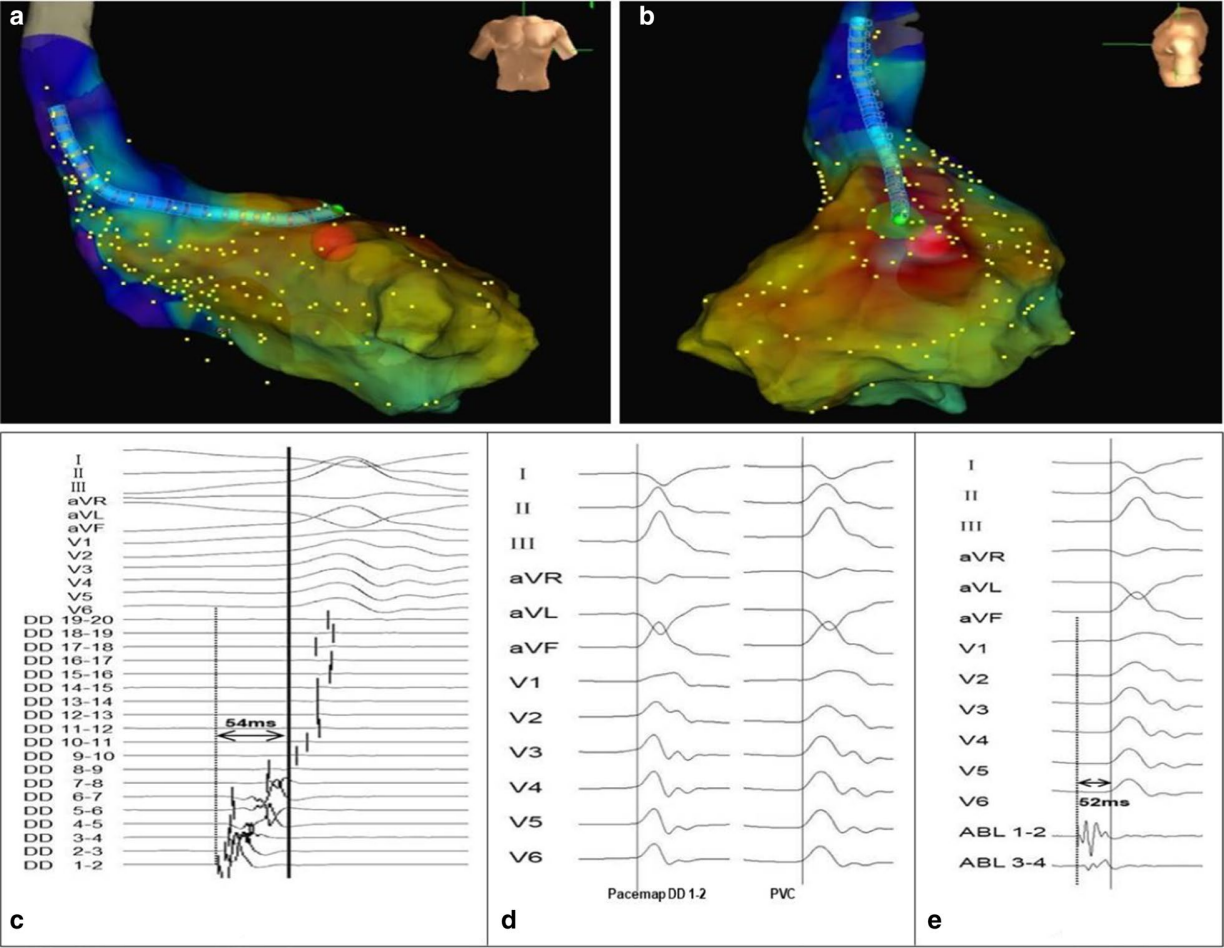
Multipolar mapping of the APM with the linear duodecapolar catheter in patient 3. **a**, **b** High-density mapping of left ventricle using the linear duodecapolar catheter showed the site of earliest ventricular activation during clinical PVCs was located at the APM, which was marked with the red dot. **c** Intracardiac electrogram during PVCs demonstrated the earliest ventricular potential recorded at DD 1–2 (54 ms pre-QRS), and they conducted upward along the linear catheter. **d** Pacing from a site with earliest ventricular activation seen on DD 1–2 revealed a perfect pace map match. **e** Radiofrequency energy was delivered to the target site of the red dot with 52 ms pre-QRS and PVCs terminated following transient monomorphic non-sustained ventricular tachycardia. DD, duodecapolar catheter. Other abbreviations as in Fig. 1

**Fig. 3:**
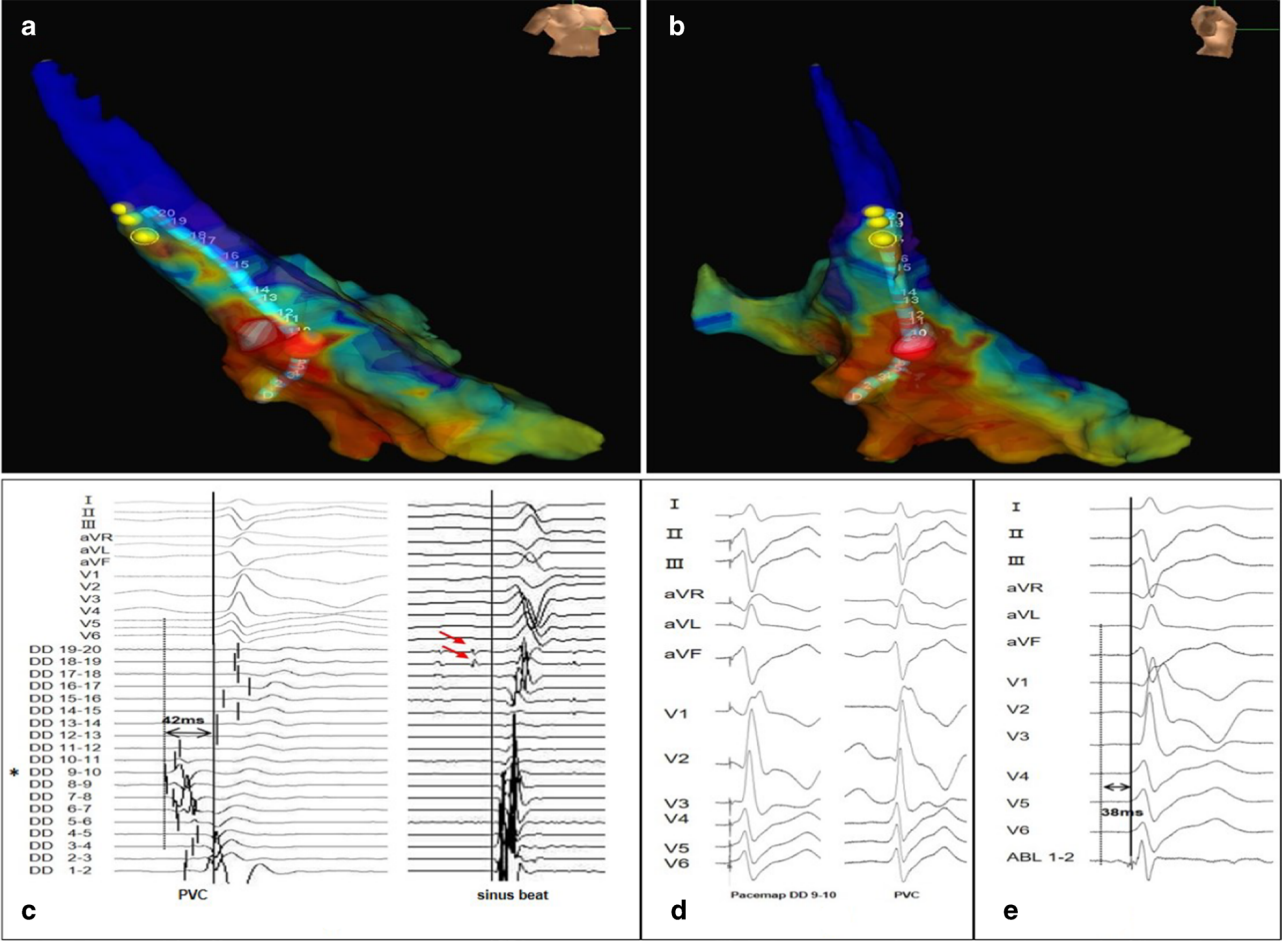
Multipolar mapping of the PPM with the linear duodecapolar catheter in patient 5. **a**, **b** A 3-dimensional ventricular activation map during clinical PVCs guided by the linear duodecapolar catheter showed ventricular excitation sites in the PPM of the left ventricle. The red dot indicated the site where the earliest ventricular excitation potential was recorded during PVCs. The yellow dots on the proximal duodecapolar catheter indicated the His-bundle site. **c** Intracardiac electrogram recorded with a duodecapolar catheter during the PVCs (left panel) showed the earliest local ventricular potential preceding the QRS onset by 42 ms located at DD 9–10 (asterisk), and they conducted upward and downward along the linear catheter, respectively. During the sinus beat (right panel), the proximal DD 19–20 and DD 18–19 (red arrow) recorded his bundle potentials. **d** Pacing from a site with earliest ventricular activation seen on DD 9–10 demonstrated a good pace map match. E. We delivered radiofrequency energy to the target site of the red dot with 38 ms pre-QRS and terminated PVCs. Abbreviations as in Fig. 1 and 2

A total of six different PM PVCs were mapped in the six patients. The sites of PVC origin included the anterolateral papillary muscle (APM) in 2 (33.3%) patients and the posteromedial papillary muscle (PPM) in 4 (66.7%) patients (Fig. 1). As mentioned, a detailed LV activation and pace map was performed in all patients via a retrograde aortic approach using a duodecapolar mapping catheter (Figs. 2, 3). During mapping, the near-field electrogram quality on the duodecapolar catheter seemed to be superior to the ablation catheter. In addition, earlier activation potentials with sharp initial signals were identified during mapping with the duodecapolar mapping catheter compared to the ablation catheter.

The procedural characteristics and ablation data are shown in Table 2. The ablation procedure was the first attempt in all patients. The mean number of endocardial mapping points in the PM region was 452 ± 88, with an average mapping time of 24.5 ± 4.2 min. The mean procedure time and fluoroscopy time was 95.8 ± 7.4 min and 14.2 ± 1.5 min, respectively. The mean number of ablation applications points was 6.8 ± 1.9 with an average overall ablation duration of 6.1 ± 3.0 min. Additionally, the values of earliest activation time relative to QRS onset during mapping were 37.8 ± 7.2 ms. Perfect/good pace maps were seen in all patients (100%) during the duodecapolar catheter mapping. There were no complications during the mapping and ablation procedures. Patients treated using the duodecapolar catheter showed 100% acute success rate and no reappearance occurred with an identical morphology in all patients following the procedures. Transthoracic echocardiography performed in all patients after the procedure showed increased echogenic appearance of the targeted ablation PM, which further confirmed the tissue edemas and lesions formed on the PMs.

**Table 2:** Procedure characteristics and ablation data

	1	2	3	4	5	6
Ablation procedure						
Total mapping points, number	310	458	512	480	556	397
Mapping time, minutes	18	25	28	26	29	21
Procedure time, minutes	95	85	100	105	100	90
Fluoroscopy time, minutes	15	13	15	14	16	12
Ablation data						
V-QRS, ms	32	35	52	34	38	36
Perfect/Good pace maps	Yes	Yes	Yes	Yes	Yes	Yes
Ablation applications, number	5	6	5	8	10	7
Total ablation duration, minutes	3	6	3	9	10	6
Average ablation output, W	35	35	30	30	35	35
Average Impedance drop, Ω	12	13	10	8	12	10
PVC morphology change during the procedure	No	No	No	No	No	No
PVC origin	PPM	PPM	APM	PPM	PPM	APM
Successful ablation	Yes	Yes	Yes	Yes	Yes	Yes
Years of follow-up, months	12	9	8	8	6	6
PVC burden,%	0.7	0.6	0.7	0.8	0.6	0.9

The data are presented as the numbers (%) or the means. V-QRS, earliest activation time relative to QRS onset during PVCs; APM, anterolateral papillary muscle; PPM, posteromedial papillary muscle

### Follow-up

After a follow-up period of 8.5 ± 2.0 months, the PVC burden in all patients decreased from 16.2 to 0.7% (P = 0.001) and all patients were not taking any medication. During reevaluation by echocardiography, no exacerbation of mitral regurgitation, and no aortic valve, cardiac structural or functional changes occurred using either method. No complications were observed at the end of follow-up.

## Discussion

Idiopathic PVCs from the PM may present in patients without structural heart disease and may play a role in triggers of NSVT, sustained recurrent VT, or even ventricular fibrillation [8, 9]. Mapping and catheter ablation of clinical PM PVCs are challenging because of the complexity of PM anatomy and their constant motion during the cardiac cycle [10]. In this study, we performed detailed activation mapping using a duodecapolar mapping catheter in order to achieve faster and more precise delineation of the site of origin of arrhythmia in a relatively small PM PVCs cohort. The most important finding of the present study is that the use of a duodecapolar catheter is feasible and highly efficient for finding the sites of earliest prepotential bipolar activity and good/perfect pace maps, leading to successful elimination of PM PVCs.

As is known, high-density mapping with multipolar catheters is a technique with proven value for activation mapping and electroanatomic substrate delineation in macroreentrant atrial tachycardia, atrial fibrillation as well as ventricular tachycardia [4, 11–13]. In the current study, to our knowledge, we are the first to describe a technique using a linear duodecapolar catheter for endocardial electroanatomic mapping and as a guide for ablation of PM PVCs. Significantly earlier activation potentials were identified during mapping with the duodecapolar mapping catheter in this study. With 2-mm interelectrode spacing, good or perfect pace maps can be generated within a given small area after high-density activation mapping with a multipolar catheter. The present results have demonstrated that patients with duodecapolar catheter mapping had short procedure and fluoroscopy times, and had short ablation duration as well as few ablation applications. Furthermore, the rate of complete PM PVCs elimination tended to be high when using multipolar catheters.

Notably, ablation of PM PVCs has a variable success rate because of challenges in mapping and catheter stability. Acute procedural success for ablation of PM PVCs is generally fair (60%-100%) and recurrence of similar morphology arrhythmias that require a repeat ablation procedure is common (approximately 5–58%) [10, 14–16]. The outcome of catheter ablation in our series was reasonably good. In our study, a higher efficiency and higher success rate when using the duodecapolar catheter may be attributed to the following aspects. Firstly, localization of PM PVCs foci relies mainly on accurate activation mapping of clinical PVCs, usually complemented with pace mapping, which is important for successful ablation of such arrhythmias. Using a 20-pole mapping catheter with 2-mm interelectrode spacing can yield a greater electrophysiologic understanding of the earliest ventricular activation areas during PVCs and therefore a more efficient targeting of arrhythmia sources and exit sites is achievable. Potential ablation targets can be identified expeditiously and an ablation catheter can be directed to these sites. A more favorable outcome using the duodecapolar catheter seems to be mediated by a more comprehensive treatment of PVCs substrate. Secondly, making adequate catheter contact of catheter tip with the contracting muscle during systole is also essential for successful ablation. Intracardiac echocardiography (ICE) could allow real-time visualization of anatomical landmarks in the PM region and ensure adequate catheter-tissue contact, which could be complementary with high-density mapping and ablation for PM PVCs. Despite an ICE-guided high-density mapping approach not being adopted in the current study cohort, it was helpful to confirm adequate contact between the ablation catheter and the endocardial surface with fluoroscopy, mapping system geometry, and the quality of local electrograms. The high mapping density created by the duodecapolar catheter and subsequent earliest activation potentials with sharp initial signals are specific for avoiding poor contact. We also found that for a given targeted area, the near-field electrogram quality on the duodecapolar catheter was often superior to the ablation catheter. This may be due to the smaller size of sensing electrode and tighter bipole spacing of the duodecapolar catheter compared to larger electrode sizes of conventional point-by-point mapping catheter. Moreover, it is not clear whether a transseptal or retrograde aortic approache is superior for ablation of PM PVCs. Published research investigating patients requiring ablation of PM PVCs tended to use the retrograde aortic approach and may offer greater catheter stability especially in the PPM while ablation for APM PVCs are better approached with a transseptal access [14, 17, 18]. In our series, PPM PVCs accounted for 66.7% of cases and adequate catheter contact by the retrograde aortic approach may be an underlying reason for the high acute ablation success rate. Finally, in patients with structurally normal hearts, the mechanism of PM PVCs tended to be focal, due to either triggered activity or enhanced automaticity [19]. The phenomenon of spontaneous PVCs with single QRS morphologies as well as lack of change in QRS morphology after ablation were seen in this cohort of patients with structurally normal heart, which is inconsistent with the literature that has reported almost half of patients with PM PVCs may exhibit spontaneous multiple QRS morphologies during an electrophysiology study [14]. Only single PVCs QRS morphologies observed in this study may lend credence to the hypothesis that PVC origin was from a single intrapapillary focus with conduction to a solitary breakout site [15], and made it possible that less extensive ablation at sites of PVC origin with excellent pace maps was successful in this study.

Apart from a duodecapolar catheter, high-density mapping catheters frequently used include the Inquiry Afocus 20-pole deflectable spiral catheter (St. Jude Medical), the Pentaray catheter (Biosense Webster, Diamond Bar, CA, USA) and the Advisor HD Grid catheter (Abbott Medical, Abbott Park, IL, USA). Koutbi et al. [19] used a 20-pole deflectable spiral catheter for ablation of PVC originating from the left ventricular PM in four patients with structural heart disease, and they found that this mapping technique was more straightforward and feasible and complete PVCs abolition was achieved for all patients. However, technically, although its rounded shape is minimally arrhythmogenic, which makes it easier to map PVCs by avoiding mechanical ectopic beats, there is the risk of the spiral being caught on the submitral apparatus and traumatizing this region of the left ventricle. Therefore, counterclockwise rotation or traction must be avoided and each time the catheter was caught in the chordae tendineae, a clockwise rotation easily extricated the spiral. Furthermore, the star shape of Pentaray catheter is more arrhythmogenic and does complicate interpretation of the activation map and can be detrimental for PVCs mapping, but has been shown to be accurate and expeditious in the mapping of complex reentrant atrial arrhythmias and atrial fibrillation [4]. The Advisor HD Grid catheter is a novel 4-by-4 unipolar electrode array with 1-mm electrodes and its splines are very pliable, which can very nicely conform to the shape of LV chamber and reduce catheter-induced ectopy [20]. Multipolar mapping with the HD Grid catheter may become an appropriate modality for PM PVCs mapping and ablation. As mentioned above, it may be advantageous to use a duodecapolar mapping catheter in the ventricle for rapid mapping and to guide ablation. On the one hand, due to the steerable and straight shape, it has less probability of arrhythmogenicity and damaging the submitral apparatus such as chordae tendineae. On the other hand, by using a 20-pole catheter with 2-mm interelectrode spacing for mapping, it can create a high sampling mapping density and multiple targets of earliest activation potentials can be identified rapidly and simultaneously. Matching pace maps can be generated within a given area with reference to the activation mapping target, and an ablation catheter can be directed to these sites previously tagged on electroanatomic mapping systems, which may significantly decrease mapping time. Finally, the increased mapping density created and high quality of the earliest activation potentials as well as near-field electrogram with sharp early signals identified by duodecapolar catheter, due to simultaneous recordings of local potential from different pairs of electrode with a relative short interelectrode distance, are specific for confirming adequate catheter contact and further ablation.

### Limitations

Firstly, the design of the study is retrospective and single-center in a relatively small sample size cohort with no structural heart disease, which was therefore subject to a myriad of biases, particularly selection bias and statistical power limitations. Hence, results from the current data need to be confirmed by further large-scale studies, which would be helpful to validate the reproducibility of the duodecapolar catheter mapping technique. Secondly, although the acute clinical success rate and the PVC burden during follow-up in our series were satisfactory, long-term outcomes are needed to demonstrate feasibility and efficacy. Moreover, the technique was only employed using single ventricular access via retrograde transaortic approach in this study, and studies on double ventricular access obtained via double transseptal or double retrograde approach may be an area of future investigation optimizing the capability to manipulate the ablation catheter to the targeted multipolar electrode. Furthermore, ICE, which may create a detailed ICE-based anatomic reconstruction of the LV and allow assurance of adequate catheter-tissue contact and optimal alignment of the catheter tip with the PM axis, was not routinely used to guide mapping and ablation, although the post procedure echocardiographic appearance in our cases confirms that the lesions have indeed been generated on the PM. Finally, an ablation catheter with contact force (CF) capabilities was not utilized in this study, which not only provides CF information but also shows vector orientation of the catheter tip.

## Conclusions

The present study provided insight into the mapping and ablation of PM PVCs using a duodecapolar catheter to perform high-density mapping of LV endocardial activation in the structurally normal heart. Multipolar mapping with a duodecapolar catheter has the potential to facilitate identification of earliest activation potentials as well as pace mapping with a high procedural success rate, and could be considered in the mapping and ablation of PM PVCs.

## Data Availability

The datasets used during the current study are available from the corresponding author on reasonable request.

## Abbreviations

PM: Papillary muscles
PVCs: Premature ventricular complexes
BMI: Body mass index
LAD: Left atrial diameter
LVEDD: Left ventricular end-diastolic diameter
LVEF: Left ventricular ejection fraction
MR: Mitral regurgitation
NSVT: Nonsustained ventricular tachycardia
CS: Coronary sinus
V-QRS: Earliest activation time relative to QRS onset during PVCs
APM: Anterolateral papillary muscle
PPM: Posteromedial papillary muscle
RBBB: Right bundle branch block
DD: Duodecapolar catheter
ICE: Intracardiac echocardiography
